# Targeted Next Generation Sequencing Revealed a Novel Homozygous *Loss-of-Function* Mutation in *ILDR1* Gene Causes Autosomal Recessive Nonsyndromic Sensorineural Hearing Loss in a Chinese Family

**DOI:** 10.3389/fgene.2019.00001

**Published:** 2019-02-05

**Authors:** Jinxia An, Jie Yang, Yan Wang, Yanxia Wang, Baicheng Xu, Guangmei Xie, Sanming Chai, Xiaoling Liu, Sijuan Xu, Xiaoxiao Wen, Qing He, Huijun Liu, Chen Li, Subrata Kumar Dey, Yali Ni, Santasree Banerjee

**Affiliations:** ^1^Gansu Provincial Maternity and Child-care Hospital, Lanzhou, China; ^2^Lanzhou University Second Hospital, Lanzhou, China; ^3^Department of Cell Biology and Medical Genetics, School of Medicine, Zhejiang University, Hangzhou, China; ^4^Department of Biotechnology, Centre for Genetic Studies, School of Biotechnology and Biological Sciences, Maulana Abul Kalam Azad University of Technology (Formerly West Bengal University of Technology), Kolkata, India

**Keywords:** hereditary hearing impairment, *ILDR1*, targeted next generation sequencing, novel mutation, Chinese population

## Abstract

Hereditary hearing impairment is one of the major and common birth defects in Chinese population. Non-syndromic sensorineural hearing loss (NSHL) is the most common types of hereditary hearing impairment. Genotypically and phenotypically NSHL is extremely heterogenous and follow either autosomal dominant or autosomal recessive or X-linked mode of inheritance. Presently, 127 genes have been identified to be associated with both syndromic and (NSHL). Here, we studied a Chinese family with moderate and profound hearing impairment. The proband is a 30-year old Chinese man. The proband was born with normal hearing and at the age of 5-years, the proband was first noticed with hearing impairment. Gradually and progressively the proband was presented with loss of hearing in his both right and left ears at the age of 30 years. The clinical symptoms, age of onset or progression to loss of hearing was similar in both the proband and his younger brother. The proband’s parents are phenotypically normal and non-consanguineous. Clinical diagnosis of the proband and his younger brother has been done by classical pure tone audiogram (PTA). Computed Tomography (CT) found no abnormality in bilateral external ear, middle ear and inner ear. Targeted next generation sequencing was performed with a panel of 127 genes reported to be associated with hereditary hearing impairment. A novel homozygous single nucleotide deletion (c.427delT) in exon 4 of *ILDR1* gene has been identified in proband and in his younger brother. Sanger sequencing confirmed that proband’s father and mother are carrying this mutation in a heterozygous manner. This mutation has not been identified in 100 normal healthy control individuals. This mutation (c.427delT) causes frameshift (p.Tyr143Ilefs^∗^19) which leads to the formation of a truncated ILDR1 protein of 162 amino acids instead of the wild type ILDR1 protein of 546 amino acids. *ILDR1* associated hereditary hearing impairment is very rare and this is the first report of identifying a *loss-of-function* mutation in *ILDR1* gene associated with hereditary hearing impairment in Chinese population. Our present study also emphasized the significance of rapid, accurate and cost-effective screening for the patient with hereditary hearing impairment by targeted next generation sequencing.

## Introduction

Hereditary hearing loss (HL) is the major and most common sensorineural disorders with an incidence of 1/1000 live birth world-wide (Aslam et al., 2005). However, hereditary HL are mostly caused by the germline mutations of a group of genes (Wang et al., 2018). Non-syndromic sensorineural hearing loss (NSHL) is the major types of all HL and more than 80% of all NSHL cases are caused by genetic alterations or pathogenic mutations (Zhao et al., 2014). Till now, more than 100 genes are identified to be associated with NSHL and more than 60 genes are associated with autosomal recessive non-syndromic sensorineural hearing loss (ARNSHL; Parker and Bitner-Glindzicz, 2015; Van Camp and Smith, 2017). ARNSHL are a group of rare, non-progressive, severe and pre-lingual form of hereditary (HL) and majorly caused by the germline mutations of *GJB2, SLC26A4*, *MYO7A, OTOF, CDH23, TMPRSS3*, and *TMC1.* Different populations identified with different founder mutations underlying the phenotype of ARNSHL (D’Adamo et al., 2009).

Recently, Borck et al. (2011) reported that *loss-of-function* mutation of *ILDR1* gene was causing very rare non-syndromic autosomal recessive deafness type 42 (DFNB42). *ILDR1* gene is located in chromosome 3. *ILDR1* gene is consisting of 8 exons. Human *ILDR1* gene encodes the immunoglobulin-like domain containing receptor 1 (ILDR1), a predicted type 1 transmembrane protein. ILDR1 protein showed a tissue specific expression and it is highly expressed in prostate, testes, pancreas, and kidney tissues. However, in mouse and zebrafish, ILDR1 is playing a major role in the development of auditory hair cells, semicircular canal and tricellular tight junction (Higashi et al., 2013; Morozko et al., 2014). *In vitro* study showed that loss of both outer and inner hair cells leads to profound sensorineural hearing loss in mouse upon complete knockout of *ILDR1* gene (Sang et al., 2014). It has been reported that IDLR1 protein may involve as barrier for cellular tight junction (Higashi et al., 2015). In addition, Kim et al. reported that mutation in *ILDR1* causes disruption of tricellulin, hence, the formation of tricellular tight junction can’t be possible (Kim et al., 2015). Association between germline mutation in *ILDR1* gene and ARNSHL has been confirmed again for patients with hearing loss in both the Saudi-Arabian and the Iranian populations (Ramzan et al., 2014; Mehrjoo et al., 2015).

However, due to extreme genotypic and phenotypic heterogeneity, genetic screening and clinical diagnosis of patient with hereditary (HL) is really very challenging. So, we customize a gene panel and performed targeted next generation sequencing for the screening of the patients with hereditary non-syndromic sensorineural hearing loss (SNHL; Diaz-Horta et al., 2012).

In our present study, we identified two patients with hereditary (NSHL). Targeted next generation sequencing and sanger sequencing identified a novel homozygous single nucleotide deletion in exon 4 of the *ILDR1* gene. *ILDR1* associated hereditary (HL) is a very rare disorder and only 20 mutations of *ILDR1* gene has been reported so far. In Chinese population, we first report the *“loss-of-function”* mutation in *ILDR1* gene causing hereditary (HL) with an autosomal recessive mode of inheritance. Our present study also describes the significance of targeted next generation sequencing for rapid, accurate and cost-effective approach for screening of the patient with hereditary hearing impairment.

## Case Report

**FIGURE 1:**
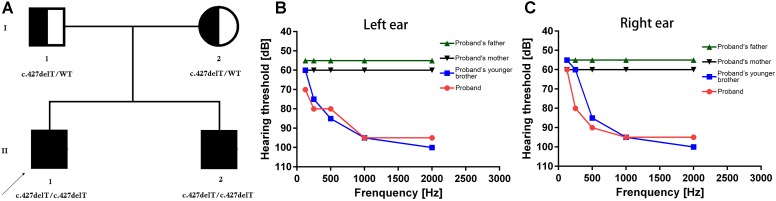
**(A)** Pedigree of the family. The filled symbol indicates the patient (proband and his younger brother), and the half-filled symbols show the carrier parents, who were heterozygous carriers but were unaffected. The arrow points to the proband. **(B,C)** Hearing threshold dynamics of PTA test across analyzed frequencies (0.25–2 kHz). A clear and gradual drop in hearing threshold from higher to lower frequency range was identified in the proband (red line) and in the proband’s younger brother (blue line) in the both **(B)** left ear and **(C)** right ear. Normal hearing in higher to lower frequency range has been identified in proband’s father (green line) and mother (black line) in both left **(B)** and right **(C)** ears.

Our present study included a Han Chinese family. The proband is a 30-year old man of non-consanguineous Chinese parents (Figure 1A). Proband (II-1) was clinically diagnosed with NSHL. Proband’s younger brother (II-2) also identified and diagnosed with NSHL. Proband’s father (I-1) and mother (I-2) are phenotypically normal. Clinical diagnosis has been done at Gansu Provincial Maternity and Child-care Hospital, LanZhou, China.

Clinical diagnosis of the proband and proband’s younger brother has been done on the basis of complete medical history, family history and detailed physical examination. Proband and his younger brother’s auditory test has been performed with otoscopic examination and pure tone audiometry (PTA) followed by the brainstem evoked response audiometry (BERA). In addition, hearing threshold of proband and his younger brother were evaluated or estimated by the air-conduction pure-tone average thresholds ranging from 250 to 8000 Hz. However, PTA, BERA and hearing threshold were also evaluated for proband’s father and mother and no abnormality was found.

In PTA test, hearing level was categorized into five groups; viz., normal (<20 dB), mild (20–40 dB), moderate (41–70 dB), severe (71–90 dB), and profound (>90dB). Here, hearing threshold was calculated based on the average of the right and left ears. In order to understand the structural abnormality of the temporal bone, computerized tomography (CT) scan was performed. Vestibular function analysis was done by caloric stimulation.

Proband and his family members denied to have any previous potential causes of acquired hearing loss. In addition, audiological history of this family has been obtained to understand the age of onset, gradual progression rate and presence of other related audiological symptoms. Four family members (proband, proband’s younger brother and their parents) underwent a clinical otorhinolaryngological examination. Moreover, tympanogram was performed and middle-ear associated causes of (HL) were excluded. In addition, other ophthalmological and pedo-neurological examinations was performed.

This study was approved by the ethics committee of the Gansu Provincial Maternity and Child-care Hospital, LanZhou, 730050, China, in accordance with the recommendations of the Declaration of Helsinki. Written informed consent has been obtained from all the participant of this study in accordance with the Declaration of Helsinki.

The proband (II-1, 30-year old) was presented with bilateral hearing loss and admitted to our hospital. Proband’s detailed and comprehensive medical report showed that there is no abnormality in vestibular organs. Caloric stimulation test also supported it. In contrast with other NSHL cases, here the proband’s speech perception ability was not impaired. PTA test result revealed that the hearing perception level were 60, 80, 90, 95, and 100 dB for the right ear and 70, 80, 85, 95, and 100 dB for left ear for stimulation at 0.125, 0.25, 0.5, 1, and 2 kHz frequencies, respectively, (Figure 1B,C). Computed tomography scan revealed no abnormality in medial and inner ear with normal temporal bone.

The proband’s younger brother (II-2, 25-year old) was presented with bilateral hearing loss and admitted to our hospital. He was not identified with any abnormality in vestibular organs. His speech perception ability was quite well. The PTA test for proband’s younger brother (II-2) revealed that the hearing threshold 55, 60, 85, 95, and 100 dB for the right ear and 60, 75, 85, 95, and 100 dB on the left ear at 0.25, 0.5, 1, 2, and 4 kHz frequency range, respectively, (Figure 1B,C). The CT scan of temporal bones was normal.

Both these patients are clinically diagnosed with (NSHL). The patient’s parents are phenotypically normal (Figure 1B,C).

## Materials and Methods

### Targeted Next-Generation Sequencing

**FIGURE 2:**
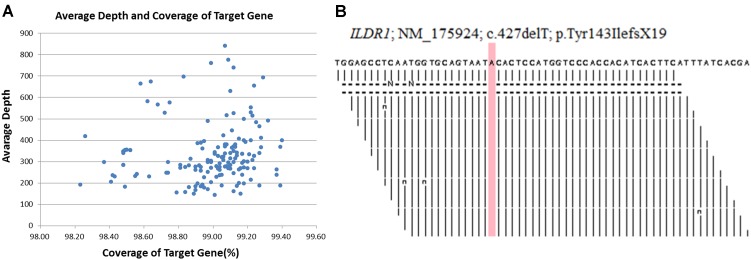
**(A)** Average depth and coverage of Target genes associated with hereditary hearing impairment. **(B)** A snapshot across ILDR1 locus showing read depth at this causal variant in BAM file.

**FIGURE 3:**
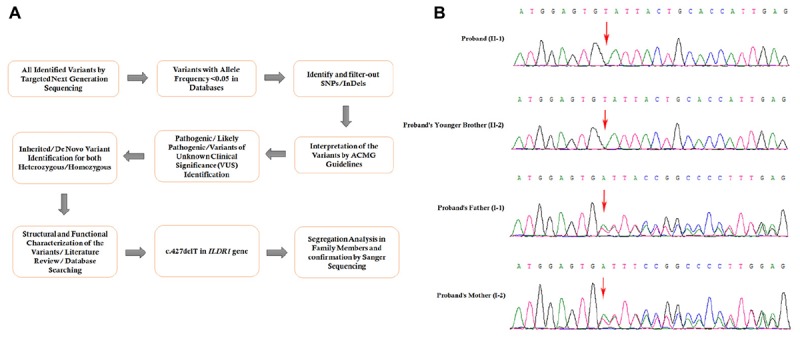
**(A)** The comprehensive and detailed method of variant interpretation. **(B)** Sanger sequencing analysis of *ILDR1* mutation in the family (sequencing is shown on complementary strand). The homozygous novel deletion (c.427delT) was observed in the proband (II:1), and proband’s younger brother (II:2). Proband’s father (I:1), and mother (I:2) were carrying the deletion with heterozygosity.

DNA samples obtained from the proband (II-1), his younger brother (II-2), his father (I-1) and his mother (I-2) were sequenced using target exome-based next-generation sequencing. Roche NimbleGen’s (Madison, United States) custom Sequence Capture Human Array was used to designed to capture targeted sequence, covering all exons and flanking sequence (including the 100 bp of introns) of 127 genes which is associated hereditary hearing impairment (Supplementary Table 1). The details of targeted next generation sequencing have been described in Supplementary Table 2. The 127-gene panel achieved a total of 619.167 kb of targeted sequence, covering 2,268 exons and flanking sequence. An average of 2022981 reads per sample was acquired, with approximately 85% mapping to their targets. The average mean depth for the targeted regions was 311.3 ± 56.7; 97.5 ± 0.1% of the covered exons had ≥ 30 reads. Average depth and coverage of Target genes has been described in Figure 2A. Figure 2B, is showing read depth at this causal variant in BAM file across *ILDR1* locus. The procedure for preparation of libraries was consistent with standard operating protocols published previously (Yang et al., 2018). According to the standard protocol, simultaneously we sequenced 30 samples on Illumina HiSeq 2500 Analyzers (Illumina, San Diego, United States) for each pooling batch for 90 cycles (specially designed rare disease screening). We applied Illumina Pipeline software (version 1.3.4) to generate the raw data which is later used for Bioinformatic analysis. We extract the clean reads from the raw reads by using already established filtering criteria (Wei et al., 2014). Then, we selectively using at least 90 bp long clean reads for aligning to the human reference genome (Build 37) of NCBI database by using Burrows Wheeler Aligner (BWA). BWA, a multi-vision software package, generating the output file in bam format. After that, target region coverage, sequencing depth, SNP/InDel/CNV detection has been analyzed by using the bam data. Next, SOAPsnp software, Sam tools pileup software and Bioinformatic computational framework were established for identifying SNP, InDels and CNVs. Filtering criteria for a SNP or InDel has been set with at least 10 reads and >20% of total reads. SNPs are filtered out and selected for further interpretation if the frequency of the SNPs is <0.05 in dbSNP, HapMap, 1000 Genomes database, the 100 healthy reference samples (same ethnic origin with similar age and sex range) sequenced in this study. The comprehensive and detailed method of variant interpretation has been described in Figure 3A.

### Sanger Sequencing

Sanger sequencing has been used to validate this homozygous mutation identified by targeted next generation sequencing by using the following primers: F-5′-AGGCGCGGAGCCTTATGCCCAG-3′, R-5′-GAACCGGCCGCTTAGGGGCCGG-3′. The reference sequence; NM_175924 of *ILDR1* was used.

### Identification of Novel Mutation in *ILDR1* Gene

Targeted next generation sequencing and Sanger sequencing identified a homozygous novel single nucleotide deletion (c.427delT) in exon 4 of *ILDR1* gene (Figure 3B). This mutation leads to formation of a truncated (p.Tyr143Ilefs^∗^19) ILDR1 protein with 162 amino acids compared with the wild type ILDR1 protein of 546 amino acids. Sanger sequencing confirmed that proband’s younger brother also carrying this homozygous mutation. The family segregation analysis was performed by sanger sequencing and identified that proband’s father and mother also carrying the heterozygous *ILDR1* c.427delT mutation. This mutation was not present in the Human Genome Mutation Database (HGMD) or in the 1000 Genomes database. This mutation was also not detected in 100 normal Chinese healthy control individuals. These findings lead us to suggest that this novel mutation found in the proband in this Chinese family may be the cause of the disease.

## Discussion

In the present study, we identified a Chinese family with (NSHL). In this Chinese family, proband and proband’s younger brother were presented with(NSHL). Targeted next generation sequencing identified a novel homozygous *loss-of-function* mutation in *ILDR1* gene in both the proband and proband’s younger brother. This homozygous mutation in *ILDR1* gene is inherited in the proband and his younger brother from their unaffected parents.

*ILDR1* associated NSHL is classified as Deafness, autosomal recessive, 42 (DFNB42). DFNB42 [MIM# 609646] is a very rare form of autosomal recessive hereditary hearing impairment (Yan et al., 2013). Previously, DFNB42 is only reported from consanguineous families, mostly from Iranian, Arabian, Turkish, Czech and Pakistani population (Borck et al., 2011; Ramzan et al., 2014; Mehrjoo et al., 2015; Bademci et al., 2016; Marková et al., 2016; Tlili et al., 2017). Recently, DFNB42 is also reported in both European and Chinese population (Liu et al., 2015; Churbanov et al., 2016). This is the first report of *loss-of-function* mutation in *ILDR1* gene associated with DFNB42 in a non-consanguineous Han Chinese family.

In our present study, targeted next generation sequencing identified a homozygous novel single nucleotide deletion (c.427delT) in *ILDR1* gene which leads to a premature stop codon and finally results in the formation of a truncated ILDR1 protein. This truncated and non-functional ILDR1 protein unable to recruit tricellulin followed by malformation of tight junctions which finally renders the normal function of auditory hair cells and causes hearing impairment (Morozko et al., 2014; Sang et al., 2014; Kim et al., 2015).

*ILDR1* associated ARNSHL is a rarest type of hereditary (HL). As it is inherited in an autosomal recessive manner, pathogenic mutation in *ILDR1* gene is causing ARNSHL either in homozygous condition or with compound heterozygosity. In addition, it has been reported that majorly *ILDR1* gene associated ARNSHL is predominantly occur in consanguineous families (Borck et al., 2011; Ramzan et al., 2014). In contrast, the segregation of the candidate mutation in this Han Chinese family apparently showed the consanguineous pattern but actually the family is truly non-consanguineous. Moreover, both the parents are carrying the same mutation in a heterozygous manner and transmit that to their next generation and offspring become the homozygous for that particular mutation and having the diseases, it is a characteristic feature of consanguineous families. Hence, in this present study, according to the segregation of the mutation among family members it follows the consanguineous pattern but actually it is a truly non-consanguineous family. So, our case is very rare, novel and unique.

Till now, only 20 *ILDR1* mutations have been reported to be associated with DFNB42 (Kovac et al., 2017; Wang et al., 2018; Table 1). DFNB42 is mostly identified and reported in consanguineous families from west and south Asia (Table 1). We reported the first *loss-of-function* mutation in *ILDR1* gene associated with DFNB42 in a non-consanguineous Han Chinese family. Among previously reported 20 mutations in *ILDR1* gene, most of them are results into the formation of a truncated ILDR1 protein (Talebi et al., 2017). Genotypically and phenotypically DFNB42 is highly heterogenous. In patients with DFNB42, age of onset and severity of hearing loss is showing extreme phenotypic heterogeneity.

**Table 1:** Summary of mutations in *ILDR1* that are associated with Deafness, autosomal recessive, 42 (DFNB42).

Mutation (cDNA)	Mutation (Protein)	Affected domains	Hearing phenotype	Ethnic group	Reference
c.3G > A	p.Met1Ileext+136	Signal peptide and extracellular domain	Moderate to profound	Pakistan	Borck et al., 2011
c.59-5_88del	p.Gly20_Thr31del	Signal peptide and extracellular domain	Moderate to profound	Iranians	Borck et al., 2011
c.82delG	p.V28Sfs^∗^31	Extracellular, transmembrane and intracellular domains	N/A	Pakistan	Borck et al., 2011
c.206C > A	p.Pro69His	Extracellular domain	Post-lingual onset and partial deafness	Korean	Kim et al., 2015
c.290 G > A	p.Arg97Gln	Extracellular domain	N/A	Pakistan	Borck et al., 2011
c.305T > A	p.Val102Glu	Extracellular domain	Severe to profound	Iranian	Mehrjoo et al., 2015
c.325_333dupAATGAGCCC	p.Asn109_Pro111dup	Extracellular domain	Moderate to profound	Saudi Arabian	Ramzan et al., 2014
c.411delG	p.Trp137Cysfs^∗^25	Extracellular domain	N/A	Pakistan	Borck et al., 2011
c.421G > C	p.Gly141Arg	Extracellular domain	Moderate to profound	Chinese	Liu et al., 2015
c.428A > G	p.Tyr143Cys	Extracellular domain	Moderate to profound	Iranian	Talebi et al., 2017
**c.427delT**	**p.Tyr143Ilefs^∗^19**	**Extracellular domain**	**Moderate to profound**	**Chinese**	**This study**
c.499+1G > A	p.Trp168Lysfs^∗^47	Transmembrane and intracellular domains	Severe	Pakistan	Borck et al., 2011
c.583C > T	p.Gln195^∗^	Intracellular domain	Severe to profound	Iranians	Borck et al., 2011
c.804del G	p.Glu269Argfs^∗^4	Intracellular domain	Severe to profound	Saudi Arabian	Tlili et al., 2017
c.820C > T	p.Q274^∗^	Intracellular domain	N/A	Iranian	Diaz-Horta et al., 2012
c.942C > A	p.C314^∗^	Intracellular domain	N/A	Iranian	Bademci et al., 2016
c.1032delG	p.Thr345Profs^∗^20	Intracellular domain	Severe	Pakistan	Borck et al., 2011
c.1135G > T	p.Glu379^∗^	Intracellular domain	Severe to profound	Pakistan	Borck et al., 2011
c.1180delG	p.Glu394Serfs^∗^15	Intracellular domain	Severe	Pakistan	Borck et al., 2011
c.1217-1218delTC	p.S406^∗^	Intracellular domain	Moderate to profound	Iranian	Mehrjoo et al., 2015
c.1358G > A	p.Arg453Gln	Intracellular domain	Severe to profound	Pakistan	Borck et al., 2011


In our present study, for identifying the candidate variants in ARNSHL family, we performed targeted next generation sequencing and confirmatory Sanger sequencing. Our study also emphasized the significance and importance of gene panel based targeted next generation sequencing for the clinical diagnosis of rare diseases. Targeted next generation sequencing is the most rapid, accurate and cost-effective approach for identifying candidate mutations for ARNSHL with extreme genotypic and phenotypic heterogeneity.

## Concluding Remarks

In conclusion, here, we studied a non-consanguineous Han Chinese family with ARNSHL. Targeted next generation sequencing and Sanger sequencing identified a homozygous novel single nucleotide deletion in *ILDR1* gene which leads to formation of a truncated ILDR1 protein. *ILDR1* associated DFNB42 is rarest form of ARNSHL. Here, we are first reporting a *loss-of-function* mutation in *ILDR1* gene associated with ARNSHL in Han Chinese population. In this study, we also emphasize the significance of targeted next generation sequencing for identifying candidate mutation in rare and highly heterogenous disorder with extreme phenotypic heterogeneity.

## Dataset With Accession Number

Database: Genome Sequence Archive, BIG Data Center in Beijing Institute of Genomics (GEO). Accession number: HRA000028. URL: http://bigd.big.ac.cn/gsa-human.

## Author Contributions

SB, CL, SD, and YN designed the project. JA, JY, XL, YW, YxW, and SC performed patient workup. XW, BX, GX, HL, SX, and QH were involved in the genetic analysis. SB, YN, CL, and SD drafted the manuscript. SB, YN, JA, and JY approved the final version to be published and agreed to be accountable for all aspects of the work.

## Conflict of Interest Statement

The authors declare that the research was conducted in the absence of any commercial or financial relationships that could be construed as a potential conflict of interest.
