# Supplementary material for: Targeted Next Generation Sequencing Revealed a Novel Homozygous Loss-of-Function Mutation in ILDR1 Gene Causes Autosomal Recessive Nonsyndromic Sensorineural Hearing Loss in a Chinese Family
Source: Front Genet. 2019 Feb 5;10:1. doi: 10.3389/fgene.2019.00001 (PMC6370629; doi:10.3389/fgene.2019.00001)
Supplement: Supplementary file 1 [file Table_1.DOCX]

**Supplementary Table 1. Hereditary hearing loss (HL) gene panel for Targeted Next-generation Sequencing.**

| Name of the disease | Genes associated |
| --- | --- |
| **Autosomal recessive nonsyndromic hearing impairment** | *GJB2, GJB6, MYO7A, MYO15A, FOXI1, KCNJ10, SLC26A4, TMIE, TMC1, TMPRSS3, OTOF, CDH23, ATP2B2, GIPC3, STRC, OTOG, USH1C, TECTA, OTOA, PCDH15, RDX, GRXCR1,*  *TRIOBP, CLDN14, MYO3A, WHRN, ESRRB, ESPN, MYO6, GJA1, HGF, ILDR1, MARVELD2, DFNB59, SLC26A5, LRTOMT, LHFPL5, BSND, MSRB3, LOXHD1, TPRN, GPSM2, PTPRQ, SERPINB6, GJB3* |
| **X-link hereditary hearing impairment** | *PRPS1, POU3F4, SMPX* |
| **Autosomal dominant nonsyndromic hearing impairment** | *ACTG1, CCDC50, CEACAM16, COCH, CRYM, DFNA5, DIABLO, DIAPH1, DSPP, EYA4, GJB2, GJB3, GJB6, GRHL2, KCNQ4, MIR96, MYH14, MYH9, MYO1A, MYO6, MYO7A, POU4F3, SIX1, SLC17A8, TECTA, TJP2, TMC1, WFS1, DIAPH* |
| **Maternally inherited hearing impairment** | *MT-RNR1, MT-TS1* |
| **Syndromic hearing impairment** | *SERAC1, PDSS1, FGFR3, FGFR1, FGFR2, PHEX, DLX5, TNFRSF11B, COL2A1, COL11A1, COL9A1, COL9A2, COL4A3, COL4A4, COL4A5, BSND, SOX9, PAX2, GATA3, SLC19A2, IGF1, PAX3, MITF, SNAI2, EDNRB, EDN3, SOX10, HOX A1, SOBP, EYA1, SIX5, SIX1, CHD7, SEMA3E,S MAD4, FGF3, TCOF1, PRRX1, GLI3, HOXA2, KCNQ1, KCNE1, CACNA1D, ALMS1, LRP2, TIMM8A, NDP, WFS1, OPA1, SLC4A11, MYO7A, USH1C, CDH23, PCDH15, USH1G, USH2A, ADGRV1, PDZD7, WHRN, CLRN1, MT-TK, MT-T E, MT-TL1, SLC26A4, KCNJ10, FOXI1* |
