# Supplementary material for: Targeted Next Generation Sequencing Revealed a Novel Homozygous Loss-of-Function Mutation in ILDR1 Gene Causes Autosomal Recessive Nonsyndromic Sensorineural Hearing Loss in a Chinese Family
Source: Front Genet. 2019 Feb 5;10:1. doi: 10.3389/fgene.2019.00001 (PMC6370629; doi:10.3389/fgene.2019.00001)
Supplement: Supplementary file 2 [file Table_2.DOCX]

**Supplementary Table 2. Description of Targeted Next generation Sequencing.**

| Panel size | 1559090 (1.56M) |
| --- | --- |
| Sample type | Whole blood |
| Quantity of DNA | ≥2ug |
| Quantity of DNA required for library construction | 1ug, 1ug more for verification by sanger or QPCR |
| Number of Pooled Hybridization | 30 at most, total 1000ng |
| Each library Pooled for hybridization | 1000ng/N, “N” is pooled number |
